# The landscape of tyrosine kinase inhibitors in sarcomas: looking beyond pazopanib

**DOI:** 10.1080/14737140.2019.1686979

**Published:** 2019-11-13

**Authors:** Christopher P Wilding, Mark L Elms, Ian Judson, Aik-Choon Tan, Robin L Jones, Paul H Huang

**Affiliations:** a Division of Molecular Pathology, The Institute of Cancer Research, London, UK; b Department of Medical Oncology, Sarcoma Unit, The Royal Marsden Hospital, London, UK; c Department of Biostatistics and Bioinformatics, Moffitt Cancer Center, Tampa, FL, USA

**Keywords:** sarcomas, kinases, tyrosine kinase inhibitors, signal transduction, targeted therapy, biomarkers

## Abstract

**Introduction**: Tyrosine kinases are key mediators of intracellular signaling cascades and aberrations in these proteins have been implicated in driving oncogenesis through the dysregulation of fundamental cellular processes including proliferation, migration, and apoptosis. As such, targeting these proteins with small molecule tyrosine kinase inhibitors (TKI) has led to significant advances in the treatment of a number of cancer types.

**Areas covered**: Soft tissue sarcomas (STS) are a heterogeneous and challenging group of rare cancers to treat, but the approval of the TKI pazopanib for the treatment of advanced STS demonstrates that this class of drugs may have broad utility against a range of different sarcoma histological subtypes. Since the approval of pazopanib, a number of other TKIs have entered clinical trials to evaluate whether their activity in STS matches the promising results seen in other solid tumors. In this article, we review the emerging role of TKIs in the evolving landscape of sarcoma treatment.

**Expert opinion**: As our biological understanding of response and resistance of STS to TKIs advances, we anticipate that patient management will move away from a ‘one size fits all’ paradigm toward personalized, multi-line, and patient-specific treatment regimens where patients are treated according to the underlying biology and genetics of their specific disease.

## 1. Introduction

Soft tissue sarcomas (STS) are a group of rare cancers that account for approximately 1% of all adult malignancies [1,2]. STS are highly heterogeneous with over 50 different histological subtypes that can occur in different anatomical locations and display vastly differing pathologies, genetic aberrations, and clinical behavior [3,4]. This heterogeneity makes STS an inherently challenging group of diseases to treat effectively.

Tyrosine kinase inhibitors (TKIs) represent the largest class of targeted therapies approved by the Food & Drug Administration (FDA) with multiple inhibitors having been licensed for the treatment of a range of different cancer types including STS [5]. For instance, imatinib is the primary treatment of patients with inoperable and advanced gastrointestinal stromal tumors (GIST) [6]. GIST is the most common subtype of STS and is characterized (in 85–90% of patients) by activating mutations in the receptor tyrosine kinases (RTKs) KIT and platelet-derived growth factor receptor (PDGFR) [7,8]. Following disease progression on imatinib, second- and third-line standard treatment in GIST utilizes the TKIs sunitinib and regorafenib, respectively [8]. Furthermore, a number of newer TKIs are at various stages of development. For instance, ripretinib, a switch control type II inhibitor of KIT, and avapritinib, a potent type I KIT/PDGFRα inhibitor, are both currently undergoing phase III trials in the third/fourth line-setting and may further improve the outcomes of patients with advanced GIST (NCT03353753, NCT03465722) [9,10]. Conversely, vandetanib, a TKI targeting vascular endothelial growth factor receptor (VEGFR) and epidermal growth factor receptor (EGFR)-dependant signaling, recently completed a phase II study in GIST patients deficient in the expression of succinate dehydrogenase (SDH), however, with no partial or complete responses observed in nine patients, the authors concluded vandetanib lacked activity in these patients [11]. Recently published preclinical work in a patient-derived xenograft model showed that SDH-deficient GIST respond to fibroblast growth factor receptor (FGFR) inhibitor monotherapy, which is further sensitized by the addition of a KIT inhibitor in combination [12]. The current gold-standard treatment paradigm for GIST, and the ongoing drive for newer agents, has been guided by the well understood underlying mechanisms of response and resistance that have been extensively described elsewhere and interested readers are directed to other reviews on this topic [6–8].

In contrast, the mechanisms of TKI response and resistance in non-GIST STS subtypes are not well understood and currently approved targeted therapies for this broad range of diseases is limited to the multi-target TKI pazopanib (Votrient®/GW786034) [5]. The approval of pazopanib in STS was based on data from the double-blind, placebo-controlled, randomized, PALETTE phase III trial (NCT00753688) that found a significant improvement in progression-free survival (PFS) in patients with non-adipocytic STS treated with pazopanib compared to placebo alone, after the failure of first- or further-line chemotherapy [13]. Notably, there was no significant overall survival (OS) benefit between pazopanib and placebo-treated patients in this trial [13]. Furthermore, clinical experience shows that a subset of patients either do not respond to pazopanib (known as intrinsic resistance) or rapidly develop acquired drug resistance upon treatment. These challenges highlight the importance of developing validated predictive biomarkers which can identify STS patients most likely to benefit from pazopanib [13,14]. Additionally, pazopanib is currently not licensed for use in liposarcomas (LPS), one of the more prevalent subtypes of STS, for which there are limited treatment options in the advanced disease setting [14,15]. In light of these challenges, there has been an ongoing effort to assess other inhibitors in the TKI class for improved efficacy in STS. The development and current clinical status of pazopanib in STS has recently been reviewed elsewhere and for the purposes of this article, we will focus on reviewing the preclinical and clinical development of other TKIs in non-GIST STS [14,15].

## 2. Preclinical characterization of TKIs

**Figure 1.**
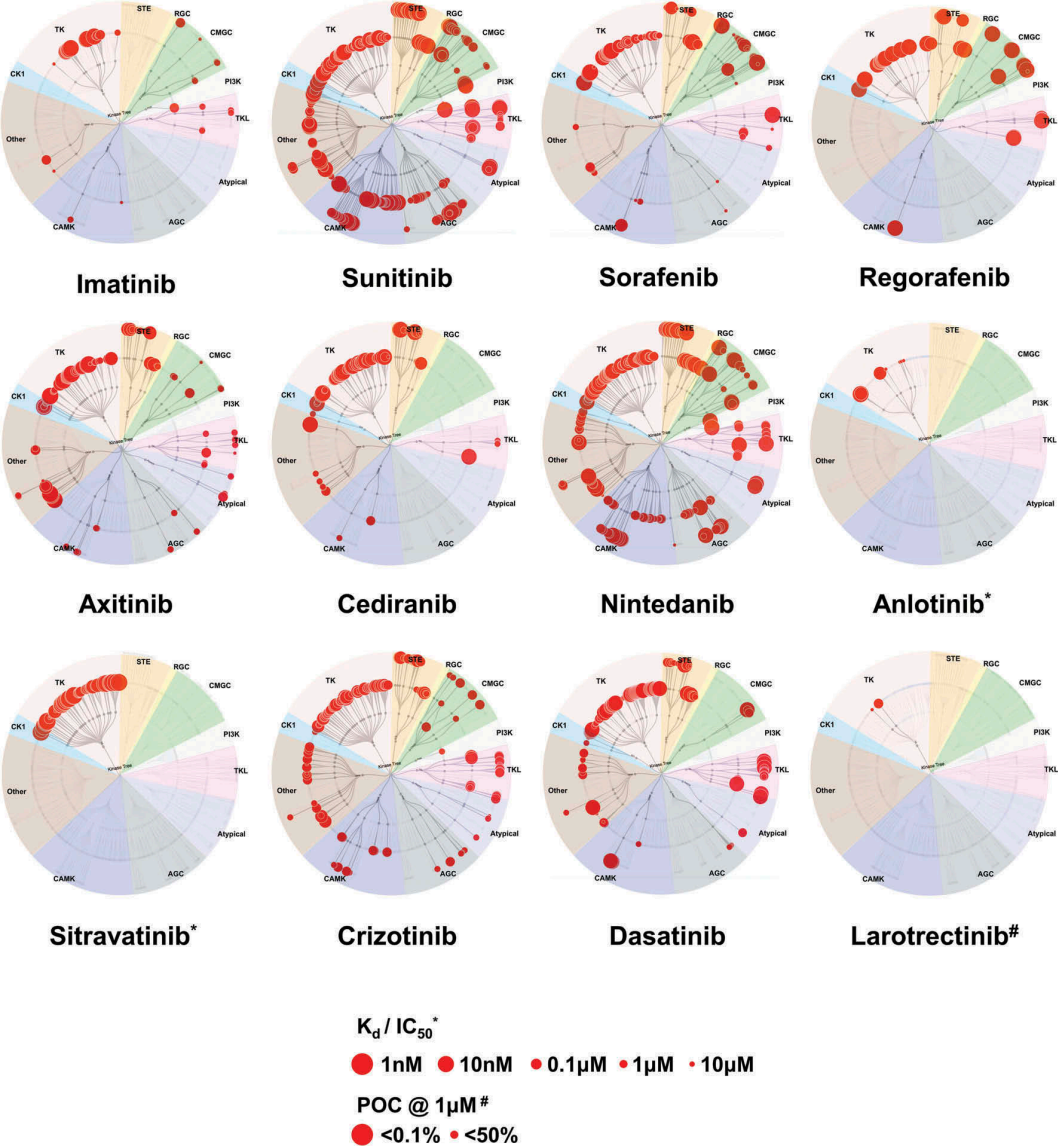
**Kinase selectivity maps**. Kinome-wide profiling measuring the dissociation constant (K_d_), inhibitory constant (IC_50_), or percent of control (POC) of the TKIs discussed within the review. The K_d_ data for imatinib, sunitinib, sorafenib, axitinib, cediranib, nintedanib, crizotinib, and dasatinib were obtained from PMID: 22037378 [16]. The K_d_ for regorafenib was obtained from PMID: 27734608 [17]. The IC_50_ for anlotinib and sitravatinib were obtained from PMID: 29446853 and PMID: 26675259, respectively [19,20]. The POC for larotrectinib was obtained from PMID: 24162815 [51]. Abbreviations: CK1; Casein kinase 1, TK; Tyrosine kinase, STE; Sterile kinase, RGC; Receptor guanylate cyclase, CMGC; Cyclin-dependent kinase, mitogen-activated protein kinase, glycogen synthase kinase, and cyclin-dependent-kinase-like kinases, PI3K; Phosphoinositide 3-kinase, TKL; Tyrosine kinase-like, AGC; Protein kinases A, G, and C, CAMK; Ca^2+^/calmodulin-dependent protein kinase.

The majority of TKIs that have shown promising preclinical and clinical efficacy in STS are multi-target TKIs that primarily target the angiogenic and growth-promoting RTKs. These RTKs include VEGFRs, PDGFRs, FGFRs, and KIT (Figure 1; Table 1) [16–26]. These TKIs are thought to exert their antitumor effects through inhibition of angiogenesis, with additional blockade of tumor growth-promoting RTKs. Examples include sunitinib, sorafenib, regorafenib, axitinib, cediranib, nintedanib, anlotinib, and sitravatinib. The preclinical characterization of these antiangiogenic TKIs have mostly followed a common drug discovery pathway starting with the identification of candidate compounds through biochemical screens of VEGFR2 kinase inhibition [20–25]. The exceptions to this are sorafenib, which was identified utilizing RAF1 kinase inhibition screens, and sitravatinib, for which preclinical characterization data are not publicly available [26]. These antiangiogenic TKIs have been found to potently inhibit VEGF-induced VEGFR2 autophosphorylation in human umbilical vein endothelial cells (HUVECs), with associated decreases in endothelial cell proliferation, migration, and endothelial tube formation [18,20,23–30].

**Table 1.** Table of tyrosine kinase selectivity of tyrosine kinase inhibitors discussed within this review.

Tyrosine kinase inhibitors	Commonly targeted tyrosine kinases in order of selectivity	References
Imatinib	**ABL1** < KIT < PDGFRB < PDGFRA (K_d_)	[16]
Sunitinib	**PDGFRB** < **KIT** < **PDGFRA** < **VEGFR2** < **VEGFR1** < RET ≪ *VEGFR3* ≪ NTRK1 ≪ ALK ≪ ABL1 < FGFR3 ≪ FGFR1/2 < NTRK2 ≪ FGFR4 = SRC ≪ NTRK3 ≪ MET (K_d_)	[16]
Sorafenib	RET < KIT < VEGFR1 < PDGFRB < *VEGFR2* < *PDGFRA* < *VEGFR3* < ABL1 ≪ NTRK3 ≪ NTRK2 ≪ FGFR2 < FGFR1 ≪ FGFR3 ≪ FGFR4 < NTRK1 (K_d_)	[16]
Regorafenib	**RET** < PDGFRB < PDGFRA < VEGFR1 < ABL1 < KIT < VEGFR3 < *VEGFR2 ≪* NTRK3 (K_d_)	[17]
Axitinib	**PDGFRA** < **PDGFRB** < **KIT** < **VEGFR1** < **VEGFR2** ≪ *ABL1* < FGFR2 < RET < VEGFR3 < FGFR3 < FGFR1 ≪ MET ≪ NTRK1 (K_d_)	[16]
Cediranib	**PDGFRB** < **KIT** < **PDGFRA** < **VEGFR1** < **VEGFR2** < **VEGFR3** < **RET** < FGFR3 < FGFR2 < *FGFR1* < *SRC* < ABL1 ≪ EGFR ≪ MET ≪ FGFR4 ≪ ALK (K_d_)	[16]
Nintedanib	**VEGFR2** < **NTRK1** < **KIT** < PDGFRB < PDGFRA < NTRK2 < ALK < RET < NTRK3 < *VEGFR1* < *FGFR1* < *FGFR3* < *VEGFR3* ≪ MET < ABL1 ≪ FGFR2 ≪ SRC ≪ FGFR4 (K_d_)	[16]
Anlotinib	**VEGFR2** < **VEGFR3** < KIT < VEGFR1 ≪ PDGFRB (IC_50_)	[20]
Sitravatinib	**VEGFR3** < **VEGFR2 = NTRK1** < **VEGFR1** = **KIT** < **NTRK2** < MET < PDGFRA < RET ≪ SRC ≪ ABL1 (IC_50_)	[19]
Crizotinib	**MET** < **ALK <** NTRK2 ≪ *ABL1* < *NTRK3* < *NTRK1* ≪ SRC ≪ RET < VEGFR1 < EGFR < FGFR3 (K_d_)	[16]
Dasatinib	**ABL1** < **SRC** < **PDGFRA** < **PDGFRB** < **KIT** ≪ EGFR ≪ RET ≪ FGFR2 ≪ VEGFR2 ≪ FGFR1 < FGFR3 ≪ VEGFR1 (K_d_)	[16]
Larotrectinib	**NTRK1 = NTRK2** ≪ *MET* < *EGFR* < *VEGFR1* = *VEGFR3* < *ABL1* = *FGFR3* < *RET* < ALK = VEGFR2 = SRC < FGFR2 < FGFR1 < PDGFRA = PDGFRB	[51]

**Key**: K_d_ or IC_50_ (x) of; **x ≤ 1 nMol, x < 10 nMol**, 10 ≤ x≤ 50 nMol, *50 ≤ x < 100* nMol, x ≥ 100 nMol. For larotrectinib, values expressed as a percent of control (POC); **x≤ 10%,** *10 ≤ x < 100*, x ≥ 100.

Abbreviations: EGFR, Epidermal growth factor receptor, FGFR; Fibroblast growth factor receptor, IC_50_; Inhibitory constant, K_d_, Dissociation constant, NTRK; Neurotrophic receptor kinase, PDGFR; Platelet-derived growth factor receptor, VEGFR; Vascular endothelial growth factor receptor.

The antiangiogenic properties of these multi-target TKIs have been further corroborated in *in vivo* murine xenograft models of varying cancer types, where drug treatment resulted in a significant reduction in microvessel area and qualitative tumor vascularity [20,23,25–34]. Furthermore, treatment of xenograft models with these TKIs commonly led to a decrease in tumor perfusion, extravasation, vascular permeability, and/or formation of metastases, thereby highlighting their antimetastatic properties [25,27,30,32,34–37]. In addition to their antiangiogenic and antimetastatic properties, these TKIs also elicited direct antitumor effects through inhibition of growth-promoting RTKs, such as PDGFRs and KIT, resulting in reductions in proliferation and migration in various tumor cell line models and bulk tumor growth in a range of xenograft models [17–37].

Other multi-target TKIs that were not developed to target the VEGFR signaling pathway have also been evaluated for the treatment of STS. These include imatinib, crizotinib, and dasatinib (Figure 1). Imatinib, crizotinib, and dasatinib were discovered through biochemical kinase screens to assess for potent inhibition of the ABL kinases, MET RTK, and Src-family kinases, respectively [38–40]. These three TKIs have been shown to exert antiproliferative and antimetastatic properties in an extensive array of *in vitro* and *in vivo* preclinical models of hematological and solid malignancies [38–49]. Additionally, in HUVEC and human lung microvascular endothelial cells, crizotinib inhibited hepatocyte growth factor (HGF)-induced MET phosphorylation and vascular tube formation [40]. Crizotinib also displayed antiangiogenic properties *in vivo* with reductions in microvessel area observed in MET-dependent murine xenografts of glioblastoma, gastric, and lung cancers [40].

More recently, highly selective TKI that target the neurotrophic receptor kinases (NTRK) have shown promising results in selected STS subtypes [50–53]. One of the most clinically advanced NTRK inhibitors is larotrectinib which inhibits all NTRK receptors at low nanomolar drug concentrations [51–53]. This inhibitor has been shown to inhibit cell proliferation and growth in *in vitro* and *in vivo* preclinical models harboring fusion NTRK oncogenes with concurrent blockade of AKT, signal transducer and activator of transcription 3 (STAT3), and/or extracellular signal-regulated kinases (ERK) downstream signaling pathways [51–53].

Building on these preclinical data, the following sections will focus on the preclinical and clinical development of these TKIs in the context of STS, as well as other clinical considerations in TKI therapy.

## 3. Histological changes associated with TKI therapy

Given the lack of “window of opportunity” studies in TKIs in sarcomas, there are only a small number of published reports of histopathological changes associated with TKI therapy. For instance, in patients with dermatofibrosarcoma protuberans (DFSP) who have undergone imatinib treatment, there is a replacement of tumor with copious amounts of hyalinized collagen, minimal necrosis, and a marked decrease in cellularity with absent mitotic figures [54]. A similar post-treatment histology is observed in GIST following imatinib therapy, characterized by extensive cystic change and hyalinization of the tumor mass [55]. Conversely, it has been reported that the use of pazopanib in infantile fibrosarcoma results in a histological response characterized by significant tumor necrosis and tumor cell death [56]. Further published descriptions of the histological effects following TKI therapy are limited to other cancer types. For example, sunitinib in the treatment of renal cell carcinoma (RCC) results in a histological response similar to that of pazopanib in infantile fibrosarcoma, characterized by extensive tumor necrosis, an associated foreign body giant-cell reaction, and absence of viable tumor [57,58]. Similarly, a complete histological response following sorafenib treatment in hepatocellular carcinoma is characterized microscopically by areas of amorphous necrosis with a surrounding fibrous capsule and complete absence of viable tumor [59]. Furthermore, as well as the histological changes reported, TKI therapy has also been associated with changes in the immunohistochemical profile observed in post-treatment tissue. For example, a case report of imatinib-treated GIST reported diffuse expression of CD117 and CD34 in the pre-treatment biopsy, which was completely absent in viable tumor areas following neoadjuvant imatinib therapy [55].

## 4. Imatinib

Imatinib (Glivec®/CGP057148B/ST-1571) was the first TKI approved for the treatment of advanced and metastatic GIST in 2002 and has been evaluated in non-GIST STS [5]. Imatinib has shown promising preclinical activity in models of malignant peripheral nerve sheath tumor (MPNST), malignant rhabdoid tumor (MRT), leiomyosarcoma (LMS), and DFSP. In MPNST cell lines, imatinib suppressed ligand-induced PDGFRβ phosphorylation and associated cellular proliferation/invasion, with a consistent phenotype also seen *in vivo* [60,61]. Imatinib has also shown antitumor effect in preclinical models of DFSP and giant-cell fibroblastoma, which are rare, recurrent, and infiltrative tumors of the dermis classically characterized by a *COL1A1*/*PDGFB* translocation [62,63]. Imatinib reduced DFSP and giant-cell fibroblastoma cellular proliferation and PDGFRβ autophosphorylation in a dose-dependent manner, with concomitant induction of apoptosis, in both *in vitro* and *in vivo* models [62,63]. Finally, imatinib has been shown to reduce *in vitro* proliferation of MRT cells, an aggressive pediatric malignancy characterized by loss of the tumor suppressor *SMARCB1*, which display constitutive ABL1 expression, as well as the SK-UT-1B LMS cell line model [64–66].

Chugh et al. reported results of their single-arm, open-label, phase II trial of imatinib in 10 histological subtypes of sarcoma (NCT00031915) (Table 2) [67]. They recruited 190 patients, of which 185 were assessable for response, and included patients older than 10 years with metastatic or locally advanced disease with a diagnosis of LMS, LPS, synovial sarcoma (SS), MPNST, fibrosarcoma, osteosarcoma, malignant fibrous histiocytoma, rhabdomyosarcoma (RMS), angiosarcoma, and Ewing’s sarcoma. There was no limit placed on number of prior therapies, with 141 (74.6%) patients having received prior doxorubicin. Patients received oral imatinib at a dose of 100mg-300mg twice a day. The primary end-point was clinical benefit rate (CBR), defined as a complete response, partial response, or stable disease, assessed on cross-sectional imaging, with an observed CBR rate of greater than 30% deemed clinically meaningful for each subtype. Across each of the subtypes assessed, a CBR of greater than 30% was not achieved in this trial, leading the authors to conclude that imatinib lacked activity in these subtypes [67]. It is interesting to note that subsequently, Chugh et al. embedded an unplanned desmoid tumor (DT) cohort in this trial and demonstrated a stable disease rate of 84% and, at 3 years follow-up, 58% of patients in this cohort were progression free [68]. DTs are a rare and locally invasive soft tissue tumor characterized by catenin beta-1 (*CTNNB1*) or adenomatous polyposis coli (*APC*) mutations. In light of these findings, subsequent phase II trials have focused their recruitment on patients with progressive DT [69,70]. Penel et al. recruited 40 patients over the age of 18 years, with proven progressive DT on cross-sectional imaging, to receive 400mg imatinib daily in a single-arm trial (NCT00287846) [69]. The primary end-point was progression arrest rate (PAR) at 3 months and the authors reported this to be 91%, with a PFS rate at 1 year of 67% and a median progression-free survival (mPFS) of 25 months. Premature drug cessation was required in 4 of the 40 patients (10%) due to the effects of drug toxicity. Kasper et al. also enrolled 38 patients with progressive DTs into a single-arm phase II study (NCT01137916) [70]. The primary end-point was progression arrest after 6 months of imatinib at a dose of 800mg daily, with the authors reporting PAR at 6 months of 65%, a rate of PFS at 1 year of 59%, and an mPFS of 21 months.

**Table 2.** Table summarizing the published results of each tyrosine kinase inhibitor discussed within this review.

	Study	Study Type	Patient Number	Chemotherapy Regimen	Subtypes (n)	Best Response	Survival
Imatinib	Chugh et al. [67]	Single arm phase II trial	190	Imatinib 300mg BD	Angiosarcoma (16)	Observed CBR 13.3%	mPFS – 2.76 months
					Ewing’s sarcoma (13)	Observed CBR 0%	mPFS – 1.68 months
					Fibrosarcoma (12)	Observed CBR 8.3%	mPFS – 1.92 months
					LMS (29)	Observed CBR 21.4%	mPFS – 2.76 months
					LPS (31)	Observed CBR 24.1%	mPFS – 3.72 months
					MFH (30)	Observed CBR 10.3%	mPFS – 1.92 months
					Osteosarcoma (27)	Observed CBR 19.2%	mPFS – 1.92 months
					MPNST (7)	Observed CBR 20%	mPFS – 1.92 months
					SS (22)	Observed CBR 15%	mPFS – 1.92 months
					RMS (2)	Observed CBR 0%	mPFS – 2.52 months
	Chugh et al. [68]	Single arm phase II trial	51	Imatinib 300mg BD	DT (51)	Stable Disease 84%	PFS at 3 years – 58%
	Penel et al. [69]	Single arm phase II trial	35	Imatinib 400mg OD	Progressive DT (35)	Complete Response 3% Partial Response 8.5% Stable Disease 80%	mPFS – 25 months
	Kasper et al. [70]	Single arm phase II trial	38	Imatinib 800mg OD	Progressive DT (38)	Partial Response 19%	PFS at 1 year – 59%
	Rutkowski et al. [71]	EORTC single arm phase II trial	16	Imatinib 400mg BD	Advanced or metastatic DFSP not amenable to curative surgery (24)	Partial Response 52.3% Stable Disease 28.6% Disease Progression 19%	PFS at 1 year – 59.7% mPFS – 20.4 months
		SWOG single arm phase II trial	8	Imatinib 400mg OD			
Sunitinib	George et al. [74]	Single arm phase II trial	53	Sunitinib 37.5mg OD	Cohort A (18) – LMS (11), SFT (3), others (4)	n/a	Stable disease at 12 weeks – 11%
					Cohort B (21) – Sarcoma NOS (5), SS (4), LPS (2), Others (10)	Partial Response 4%	Stable disease at 12 weeks – 19%
					Cohort C (9) – Chordoma (9)	n/a	Stable disease at 12 weeks – 44%
	Jo et al. [75]	Single arm phase II trial	19	Sunitinib 37.5mg OD	DT (19)	Partial Response 26.3% Stable Disease 42.1%	Median duration of response – 8.2 months PFS at 2 years – 74.7%
	Stacchiotti et al. [76]	Retrospective case series	9	Sunitinib 37.5mg OD	Progressive or metastatic ASPS (9)	Partial Response 55% Stable Disease 33%	mOS – 19 months mPFS – 17 months
	Jagodzinska-Mucha et al. [77]	Retrospective case series	15	Sunitinib 37.5mg OD	Metastatic ASPS (15)	Partial Response 40% Stable Disease 53%	mOS – 56 months mPFS – 19 months
	Stacchiotti et al. [78]	Retrospective case series	31	Sunitinib 37.5mg OD	Progressive SFT (31)	Partial Response 6.5% Stable Disease 51.6%	mPFS – 6 months
	Stacchiotti et al. [80]	Retrospective case series	10	Sunitinib 37.5mg OD	Metastatic extraskeletal myxoid chondrosarcoma (10)	Partial Response 60% Stable Disease 20% Progressive Disease 20%	mPFS not reached at median follow-up – 8.5 months
Sorafenib	Ray-Coquard et al. [89]	Single arm phase II trial	41	Sorafenib 400mg BD	Superficial angiosarcoma (26)	Complete Response 5% Partial Response 5% Stable Disease 20%	mPFS – 1.8 months
					Visceral angiosarcoma (15)	Partial Response 15.4% Stable Disease 30.8%	mPFS – 3.8 months
	Gounder et al. [96]	Retrospective case series	26	Sorafenib 400mg OD	Aggressive DT (26)	25% Partial Response 70% Stable Disease	Median time to response – 10 months mPFS not reached with median follow-up – 6 months
	Gounder et al. [97]	Phase III trial	87	2:1 randomization to placebo or sorafenib 400mg OD	Aggressive DT (87)	Complete Response 2% Partial Response 30.6%	PFS at 1 year – 89% Hazard ratio for progression or death vs placebo – 0.13 (p < 0.0001)
Regorafenib	Mir el al. [99]	Placebo-controlled phase II trial	182	1:1 Randomization to placebo or regorafenib 160mg OD	LPS (43)	Stable Disease 45% Progressive Disease 55%	mPFS – 1.0 months vs 1.7 months in placebo (p = 0.70)
					LMS (56)	Stable Disease 86% Progressive Disease 11%	mPFS – 3.7 months vs 1.8 months in placebo (p = 0.0045)
					SS (27)	Partial Response 8% Stable Disease 77% Progressive Disease 15%	mPFS – 5.6 months vs 1.0 months in placebo (p < 0.0001)
					Other sarcomas (56)	Partial Response 11% Stable Disease 67% Progressive Disease 22%	mPFS – 2.9 months vs 1.0 months in placebo (p < 0.0061)
Axitinib	Stacchiotti et al. [101]	Single arm phase II trial	17	Axitinib 5mg BD	Advanced and progressive SFT (17)	Partial Response 41.2% Stable Disease 35.3%	mPFS – 5.1 months
Cediranib	Kummar et al. [106]	Single arm phase II trial	46	Cediranib 30mg OD	Metastatic, unresectable ASPS (46)	Partial Response 35%	Disease control at 6 months – 84%
						Stable Disease 60%	
	Judson et al. [114]	Placebo-controlled phase II trial	48	2:1 randomization to placebo or cediranib 30mg OD	Metastatic, progressive ASPS (48)	Partial Response 19.4% Stable Disease 39.3%	Best median % change in sum of diameters of target lesion −15.7% vs + 1.2% in placebo (p < 0.0001) PFS at 12 months – 38.7%
Anlotinib	Chi et al. [122]	Single arm phase II trial	166	Anlotinib 12mg OD	LPS (13)	Partial Response 7.7%	mPFS −5.6 months
					LMS (26)	Partial Response 7.7%	mPFS – 11 months
					SS (47)	Partial Response 17%	Mpfs – 7.7 months
					Fibrosarcoma (18)	Partial Response 11.1%	mPFS – 5.6 months
					UPS (19)	Partial Response 5.5%	mPFS – 4.1 months
					ASPS (13)	Partial Response 46.2%	mPFS – 21 months
					CCS (7)	Partial Response 14.3%	mPFS – 11 months
					Others (23)	Partial Response 0%	mPFS – 2.8 months
Crizotinib	Schöffski et al. [130]	Single arm phase II trial	45	Crizotinib 250mg BD	Advanced or metastatic ASPS (45)	Partial Response 4.4% Stable Disease 86.7%	mPFS – 8.1 months
	Schöffski et al. [131]	Single arm phase II trial	19	Crizotinib 250mg BD	Advanced or metastatic ALK-positive IMT (12)	Objective Response 50%	PFS at 1 year – 73.3%
					Advanced or metastatic ALK-negative IMT (7)	Objective Response 14%	PFS at 1 year – 53.6%
	Schöffski et al. [132]	Single arm phase II trial	26	Crizotinib 250mg BD	Advanced or metastatic CCS with MET activation (26)	Partial Response 3.8% Stable Disease 65.4%	mPFS – 4.4 months
Dasatinib	Schuetze et al. [142]	Single arm phase II trial	109	Dasatinib 100mg BD	ASPS (12)	Choi ORR 8%	mPFS per Choi – 11 months
					Chondrosarcoma (33)	Choi ORR 15%	mPFS per Choi – 5.5 months
					Chodroma (32)	Choi ORR 19%	mPFS per Choi – 6.3 months
					ES (7)	Choi ORR 29%	mPFS per Choi – 7.9 months
					SFT (25)	Choi ORR 20%	mPFS per Choi – 2 months

Abbreviations: ASPS; Alveolar soft part sarcoma, BD; Bis die (twice daily), CBR; Clinical benefit rate, CCS; Clear cell sarcoma, DT: Desmoid tumor, ES; Epithelioid sarcoma, IMT; Inflammatory myofibroblastic tumor, LMS; Leiomyosarcoma, LPS; Liposarcoma, MFH ; Malignant fibrous histiocytoma, mOS; Median overall survival, mPFS; Median progression-free survival, MPNST; Malignant peripheral nerve sheath tumor, NOS; Not otherwise specified, OD; Omne die (once daily), ORR; Overall response rate, PFS; Progression-free survival, RMS; Rhabdomyosarcoma, SFT; Solitary fibrous tumor, SS; Synovial sarcoma, UPS; Undifferentiated pleomorphic sarcoma.

The pooled results of two separate phase II trials of imatinib in DFSP have also been reported [71]. Conducted by the Southwest Oncology Group (SWOG) (SWOG-S0245, NCT00084630) and European Organization for Research and Treatment of Cancer (EORTC) (EORTC-62,027, NCT00085475), the two trials were single-arm, single-agent, open-label, phase II trials aiming to recruit approximately 40 patients. Due to slow accrual, and following regulatory body approval of imatinib in DFSP, the trials were closed before the target recruitment was met and, as a result, the data were pooled to provide greater numbers for outcome analysis. Patients aged over 18 years with advanced or metastatic DFSP not amenable to surgery with curative intent were included, with the SWOG trial additionally including those patients in whom R0 resection was not feasible with acceptable functional or cosmetic outcomes. *PDGFB* rearrangement was confirmed in the EORTC trial by fluorescence in-situ hybridization (FISH) analysis and in the SWOG trial by reverse transcription-polymerase chain reaction (RT-PCR). A total of 16 patients were enrolled onto the EORTC trial and 8 onto the SWOG trial. The best observed response rate in evaluable patients per response criteria in solid tumors (RECIST) in pooled analysis was a partial response in 11 of 21 patients (52.3%), stable disease in 6 of 21 (28.6%), and progressive disease in the remaining 4 patients (19%). Median time to progression across the two trials was 1.7 years, with a 1-year progression-free rate of 59.7% in evaluable patients. The safety profile of imatinib across the two trials was similar to previous studies, with adverse events generally mild to moderate and easily managed with dose reduction, interruption, or supportive medical therapy. A single patient experienced grade 4 toxicity effects of thrombocytopenia and aspartate transaminase elevation, but on a background of a past medical history of pre-existing liver disturbances associated with alcohol excess. In a sarcoma subtype known to be resistant to established systemic therapies, these data demonstrate the role of imatinib as a salvage therapy in unresectable DFSP [72].

Although the initial phase II trial reported by Chugh et al. showed little in the way of promising antitumor efficacy in multiple sarcoma subtypes, subsequent studies have demonstrated the role imatinib can play in the treatment paradigm of inoperable DFSP and in actively progressive or symptomatic DT.

## 5. Sunitinib

In 2006, sunitinib (Sutent®/SU11248) was approved for the treatment of advanced GIST, following disease progression with imatinib. This drug has shown promising preclinical efficacy in certain subtypes of STS such as MRT, MPNST, and LMS [5,64,65]. In a panel of 14 cell lines consisting of differing STS subtypes, only the MRT cell lines A204 and G402 displayed sensitivity to sunitinib [65]. Consistent with this data, sunitinib treatment resulted in decreases in the phosphorylation of PDGFRα and downstream signaling node AKT [65]. In addition, small interfering RNA (siRNA)-knockdown of PDGFRα was found to phenocopy the antiproliferative effects of sunitinib and decrease cell viability in MRT cells [65]. In another study, sunitinib demonstrated antiproliferative effects only in the SK-UT-1B LMS and ST8814 MPNST cells across a panel of sarcoma cell lines [64]. Conversely, in a xenograft model of solitary fibrous tumor (SFT), sunitinib displayed only modest tumor growth inhibition when compared to another TKI regorafenib [73]. This preclinical data suggests that regorafenib is likely to be a superior choice for the treatment of SFT compared with sunitinib.

Sunitinib has been evaluated in a number of clinical trials in non-GIST STS (Table 2). George et al. reported a multicenter, single-arm, phase II study of sunitinib in metastatic or locally advanced non-GIST STS (NCT00474994) [74]. They enrolled 53 patients over the age of 18 years, of which 48 were eligible for response assessment, into three cohorts; cohort A consisting of patients with sarcoma subtypes previously shown to demonstrate response to kinase-targeted agents, cohort B consisting of subtypes with previously demonstrated inactivity to kinase-targeted agents, and cohort C consisting of patients with chordomas. A maximum of three prior lines of cytotoxic therapy was permitted, although exposure to prior sunitinib or other investigational agents was a criterion for study exclusion. When evaluated using RECIST, mPFS was 1.8 months, with 11 of 48 patients (22%) having stable disease at 12 weeks and 7 patients (14%) maintaining stable disease after 24 weeks of treatment. Given the similarities in the survival and response data of this phase II study with the PALETTE trial, in which the placebo arm had a similar mPFS of 1.6 months and stable disease as best response in 38% of the patients, it remains to be established if sunitinib is an active agent in non-GIST STS [13].

A further small, non-randomized, open-label, prospective, phase II trial of sunitinib has been undertaken by Jo et al. in which 19 patients with advanced DTs not amenable to surgery with curative intent were recruited (Table 3) [75]. Patients who had received prior arms of therapy were included in the study; four of the 19 patients (21.1%) had received prior systemic therapy, 5 of 19 (26.3%) had received prior surgery, and 4 of 19 (21.1%) had received both prior systemic therapy and surgical management. Following treatment with 37.5mg sunitinib once daily, 5 patients (26.3%) were observed to have a partial response, including response in one patient that was significant enough to enable complete resection, and a further 8 patients (42.1%) had stable disease. It should be noted that in this trial, potentially due to the prevalence of mesenteric DTs (12 out of 19), there was a high rate of serious adverse effects likely related to tumor necrosis in close proximity to the small and large bowel and the mesenteric vasculature. Of the 19 patients, one experienced an ileal perforation, one experienced a fistulous tract forming between the tumor and bowel, and there was a further episode of mesenteric bleeding.

**Table 3.** Table summarizing the clinical trials of tyrosine kinase inhibitors presented by specific soft tissue sarcoma subtype.

	TKI	Study	Study Type	Patient Number	Chemotherapy Regimen	Best Response	Survival
DESMOID TUMORS	**Imatinib**	Chugh et al.[68]	Single arm phase II trial	51	Imatinib 300mg BD	10% Progressive Disease 84% Stable Disease 6% Not Evaluable	PFS at 1 year – 66% PFS at 3 years – 58%
		Penel et al. [69]	Single arm phase II trial	35	Imatinib 400mg OD	8.5% Progressive Disease 80% Stable Disease 3% Complete Response	Median follow-up – 34 months mPFS – 25 months
		Kasper et al. [70]	Single arm phase II trial	38	Imatinib 800mg OD	19% Partial Response	PFS at 1 year – 59%
	**Sunitinib**	Jo et al.[75]	Single arm phase II trial	19	Sunitinib 37.5mg OD	15.8% Progressive Disease 42.1% Stable Disease 26.3% Partial Response	Median duration of response – 8.2 months Median follow-up – 20.3 months PFS at 2 years – 74.7%
	**Sorafenib**	Gounder et al. [96]	Retrospective case series	26	Sorafenib 400mg OD	5% Progressive Disease 70% Stable Disease 25% Partial Response	Median time to response – 10 months Median follow-up – 6 months mPFS – not reached
		Gounder et al. [97]	Phase III trial	50	Sorafenib 400mg OD	30.6% Partial Response 2% Complete Response	PFS at 1 year – 81% Median time to response – 9.6 months
				37	Placebo	20% Partial Response	PFS at 1 year – 36%
SOLITARY FIBROUS TUMORS	**Sunitinib**	Stacchiotti et al. [78]	Retrospective case series	31	Sunitinib 37.5mg OD	42% Disease Progression 51.6% Stable Disease 6.5% Partial Response	mPFS – 6 months
	**Axitinib**	Stacchiotti et al. [101]	Single arm phase II trial	17	Axitinib 5mg BD	Partial Response 41.2% Stable Disease 35.3%	mPFS – 5.1 months
	**Dasatinib**	Schuetze et al. [142]	Single arm phase II trial	25	Dasatinib 100mg BD	Choi ORR 20%	mPFS per Choi – 2 months
ALVEOLAR SOFT PART SARCOMA	**Sunitinib**	Stacchiotti et al. [76]	Retrospective case series	9	Sunitinib 37.5mg OD	Partial Response 55% Stable Disease 33%	mOS – 19 months mPFS – 17 months
		Jagodzinska-Mucha et al. [77]	Retrospective case series	15	Sunitinib 37.5mg OD	Partial Response 40% Stable Disease 53%	mOS – 56 months mPFS – 19 months
	**Cediranib**	Kummar et al. [106]	Single arm phase II trial	46	Cediranib 30mg OD	Partial Response 35% Stable Disease 60%	Disease control at 6 months – 84%
		Judson et al. [114]	Placebo-controlled phase II trial	48	2:1 cediranib 30mg OD to placebo	Partial Response 19.4% Stable Disease 39.3%	Best median % change in sum of diameters of target lesion −15.7% vs + 1.2% in placebo (p < 0.0001)
							PFS at 12 months – 38.7%
	**Anlotinib**	Chi et al. [122]	Single arm phase II trial	13	Anlotinib 12mg OD	Partial Response 46.2%	mPFS – 21 months
	**Crizotinib**	Schöffski et al. [130]	Single arm phase II trial	45	Crizotinib 250mg BD	Partial Response 4.4% Stable Disease 86.7%	mPFS – 8.1 months
	**Dasatinib**	Schuetze et al. [142]	Single arm phase II trial	12	Dasatinib 100mg BD	Choi ORR 8%	mPFS per Choi – 11 months

Abbreviations: BD; Bis die (twice daily), mOS; Median overall survival, mPFS; Median progression-free survival, OD; Omne die (once daily), ORR; Objective response rate, PFS; Progression-free survival, TKI; Tyrosine kinase inhibitor.

Further published evidence of sunitinib is limited to smaller, often retrospective case series in subtype-specific patient groups. Stacchiotti et al. have reported the role of sunitinib in alveolar soft part sarcoma (ASPS) and SFT, separately, with varying evidence of antitumor effect (Table 3). In 9 patients with progressive/advanced ASPS treated with sunitinib, 5 (55%) patients had a partial response based on RECIST, and a further 3 (33%) had stable disease [76]. Jagodzinska-Mucha et al. demonstrated a similar degree of efficacy, enrolling 15 patients with metastatic ASPS, with 6 patients (40%) observed to have a partial response to treatment and 8 (53%) with stable disease [77]. However, in 31 patients with progressive advanced SFT treated with sunitinib, of which 25 patients were pre-treated with conventional chemotherapeutic regimens, disease control was only achieved in 18 of 31 patients (58%) with a mPFS of 6 months [78]. These results are inferior to a previously published retrospective case series by Khalifa et al. of advanced SFT response to trabectedin. All of these patients received trabectedin following failure of first-line chemotherapy and the authors reported a mPFS of 11.6 months and a CBR of 81.8% [79]. Stacchiotti et al. have also reported their experience in cases of extraskeletal myxoid chondrosarcoma, which is another malignancy with an indolent natural history but with frequent metastases and known to be poorly responsive to cytotoxic chemotherapy. In their retrospective case series of 10 patients treated with sunitinib, 6 out of 10 patients (60%) had a partial response per RECIST, 2 patients had stable disease (20%), and 2 patients had disease progression on sunitinib (20%) [80].

The single-arm, non-randomized design of these studies limit any definitive conclusions regarding the efficacy of sunitinib in STS. However, the activity in specific subtypes such as SFT, extraskeletal myxoid chondrosarcoma, and ASPS are very promising despite the often indolent nature of these tumors [81–83]. Of note, there have been promising responses observed in these sarcoma subtypes traditionally resistant to chemotherapy, thereby offering salvage options in these hard to treat cases [76,77,80].

## 6. Sorafenib

Sorafenib (Nexavar®/BAY 43–9006) is another multi-target TKI, with additional activity against the RAF family kinases, currently undergoing evaluation for use in STS. Preclinically, in primary cell models of DT, sorafenib diminished cell proliferation, migration, and invasion [84,85]. These phenotypes were accompanied by a reduction in ERK, AKT, and MEK signaling with a concurrent reduction in total MEK expression [85]. Similar effects were observed in MPNST and RMS cell line models, with suppression of cell growth and associated decreases in ERK, AKT, and MEK phosphorylation [86–88]. Additionally, in the MPNST cell lines, sorafenib treatment induced G_1_ cell cycle arrest through reduction in both cyclin D1 expression and retinoblastoma protein phosphorylation [88]. Furthermore, in xenograft models of alveolar rhabdomyosarcoma (aRMS), sorafenib significantly decreased tumor growth, cell proliferation, and vascularity, accompanied by an increase in tumor necrosis [86,87]. Finally, sorafenib also displayed potent antiproliferative effects in cell line models of SFT, MRT, and LMS, with the deactivation of PDGFR signaling observed in the SFT model [64,73].

The clinical efficacy of sorafenib in STS has been evaluated in a study undertaken by the French Sarcoma Group in various vascular sarcoma subtypes (Table 2). In a single-arm, phase II study of sorafenib in angiosarcoma (NCT00874874), patients were stratified based upon the location of the tumor being either superficial (26 patients) or visceral (15 patients), with 37 (73%) patients pre-treated with conventional chemotherapy. The results were somewhat disappointing, with PFS of only 1.8 months in the superficial angiosarcoma cohort and 3.8 months in the visceral group [89]. These results are comparable to a previously published retrospective case series of a variety of second-line therapies following the failure of first-line cytotoxic regimens in metastatic angiosarcoma, which reported a median time to progression of 3.7 months [90].

In the same French Sarcoma Group trial, 5 patients with progressive SFT were included and 2 of the 5 patients (40%) achieved disease control for a period of 9 months despite having tumor progression in the month prior to commencing sorafenib [91]. Although this study showed some promising antitumor activity in SFT, the small cohort size in this study remains a limitation and larger patient cohorts are required to objectively evaluate the efficacy of sorafenib in advanced SFTs.

A further cohort of 15 patients with metastatic or locally advanced epithelioid hemangioendothelioma (EHE) not amenable to curative resection were enrolled onto this trial [92]. PFS at 9 months was chosen as the primary end-point given the indolent nature of EHE [93]. Seven of the 15 patients (46%) had undergone previous surgery and 5 patients (33%) had received prior systemic anticancer therapy. mPFS was 6 months, with a non-progression rate at 9 months of 30.7% (4 of 13 assessable patients). Best response rate on cross-sectional imaging per RECIST following sorafenib was a partial response in 2 of 13 assessable patients (13.3%) and stable disease in 9 of 13 (69.2%). In the French Sarcoma Group study, a sorafenib dose reduction was required in 3 of 15 patients (20%), whilst 5 patients (33.3%) required a transient drug discontinuation due to toxicity.

As part of these studies, circulating biomarkers for sorafenib response in the EHE and the angiosarcoma cohorts were analyzed [94,95]. Serum samples were collected at baseline and at Day 7 following commencement of treatment, with samples available for analysis from 32 patients in the angiosarcoma cohort and 13 patients from the EHE cohort. The authors reported a significant increase in the level of VEGF-A following treatment with sorafenib, with low levels of VEGF-A at baseline associated with best objective response (p = 0.04) and non-progression at 180 days (p = 0.03).

Gounder et al. performed a retrospective analysis of a case series of 26 patients with aggressive DTs treated with sorafenib. The authors reported 6 of 24 evaluable patients (25%) had a partial response to treatment and a further 17 patients (70%) had stable disease as best response (Table 3) [96]. This retrospective case series formed the basis for the subsequent double-blind phase III ALLIANCE A091105 trial of sorafenib vs. placebo in patients with DTs not amenable to surgical intervention (NCT02066181) [97]. Eighty-seven patients deemed inoperable and with proven radiographic progression were recruited and randomized to sorafenib at a starting dose of 400mg once daily or placebo at a 2:1 ratio. Aside from the absence of previous sorafenib exposure, there was no restriction on previous lines of treatment and of the 50 patients in the sorafenib cohort, 23 (46%) had previously undergone surgical resection and 18 (36%) had previously received other systemic therapy. Of the 87 patients enrolled, 84 patients were included in the analysis of response rates and primary/secondary end-points. The primary end-point of the trial was PFS and the authors reported a PFS rate after two years in the sorafenib group of 81%, compared to 36% in the placebo group (hazard ratio for progression or death 0.13, p < 0.001). An objective response per RECIST was observed in 33% of the sorafenib group (1 complete response and 15 partial responses in the 49 patients) and in 20% of the placebo group (7 partial responses in the cohort of 35). Of note, the median time to response to sorafenib was 9.6 months, which is relatively long for a TKI. OS data for this trial has not been reported. Grade 3 adverse events occurred in 14 of the 49 patients (29%) in the sorafenib arm. Dose interruptions were necessary in 65% of patients in the sorafenib arm and, as a result of adverse events, 20% of patients in the sorafenib group discontinued the trial protocol compared to none in the placebo arm.

This study is the only phase III trial of a systemic treatment that has been conducted in DTs to date and was able to demonstrate the efficacy of sorafenib to achieve durable clinical responses in this sarcoma subtype. The response rates observed in the placebo group support the role of active surveillance as the initial management for the majority of patients with DT. However, in patients with aggressively expanding or symptomatic DTs not amenable to surgical resection, the trial by Grounder et al. is potentially practice changing and has identified sorafenib as a valuable systemic treatment option in this clinical setting.

## 7. Regorafenib

Regorafenib (Stivarga®/BAY 73–5406) is a near-identical analogue of sorafenib with similar kinase selectivity and differs by the addition of one fluorine atom on the central aromatic ring [17,18,26]. As with sorafenib, regorafenib has shown promising results in preclinical STS models of MRT, LMS, and SFT [31,64,98]. In MRT, regorafenib significantly reduces cell viability in the A204 MRT cell line [31,64]. Teicher et al. reported a similar phenotype in the SK-UT-1B LMS cell line upon treatment with regorafenib [64]. When assessed in a number of SFT xenograft models, regorafenib was found to have the greatest antitumor effect in a panel of antiangiogenic TKIs and bevacizumab – a humanized therapeutic antibody that binds circulating VEGF and blocks the ligand from binding to VEGFR [73,98]. Immunoblotting analysis of these xenograft tumors 4 weeks post-treatment found that regorafenib led to decreases in PDGFRβ and VEGFR2 phosphorylation, whereas the rest of the TKI panel inhibited only either one or none of these targets, thereby explaining the greater effect of regorafenib in SFT [73].

Regorafenib was evaluated in STS in the REGOSARC trial (NCT01900743) [99]. This randomized, placebo-controlled, double-blind, phase II clinical trial was undertaken by a French-Austrian collaborative and enrolled patients aged over 18 years with advanced STS pre-treated with doxorubicin or any other anthracycline-based therapy. Patients were randomized 1:1 into either the placebo or the regorafenib arm and stratified based on sarcoma histological subtype into one of the four cohorts: LPS, LMS, SS, or other sarcomas. When compared with placebo, regorafenib induced significantly prolonged mPFS in the LMS subgroup (3.7 months vs 1.8 months, p = 0.0045), the SS subgroup (5.6 months vs 1.0 months, p < 0.0001), and in the other sarcomas subgroup (2.9 months vs 1.0 months, p = 0.0061). However, regorafenib failed to demonstrate efficacy in the LPS cohort with a worse mPFS compared to placebo (1.0 months vs 1.7 months, p = 0.70). These data represent the most compelling evidence thus far for the use of regorafenib in the treatment of non-adipocytic STS. Unfortunately, as was the case in the PALETTE trial, this improvement in mPFS was not translated into a significant improvement in OS in any of the four subtype cohorts (Table 2) [13]. Based on these results, regorafenib warrants further evaluation in STS and investigation of potential molecular biomarkers that may stratify patients and identify those most likely to gain OS benefit from this drug. Identification of such predictive biomarkers for benefit from regorafenib would facilitate rational patient selection in future clinical trials.

## 8. Axitinib

Preclinical studies of axitinib (Inlyta®/AG013736) in STS have reported efficacy in models of myxoid LPS; an STS subtype for which there are currently no approved TKIs [100]. In a screen of 43 drugs, axitinib was found to strongly inhibit the growth of patient-derived myxoid LPS cell lines and xenografts, with an observed reduction in the phosphorylation of KIT, VEGFR3, PDGFRβ, and downstream signaling proteins AKT and ERK [100]. Furthermore, axitinib was also found to repress VEGFR1 and VEGFR3 as well as VEGFA and VEGFB gene expression [100]. Consistent with this antiangiogenic activity, addition of conditioned media from myxoid LPS cells treated with axitinib to HUVECs reduced endothelial tube formation compared to conditioned media from vehicle-treated cells [100]. In these myxoid LPS models, axitinib treatment led to G_1_ phase cell cycle arrest and induced cell death [100]. In addition to activity against myxoid LPS, axitinib has also shown potent antiproliferative effects in MRT, LMS, and SS cell lines [64].

Axitinib has been evaluated in a phase II clinical trial in progressive and advanced SFT (NCT02261207) [101]. In this study, 17 patients with advanced SFT, with evidence of progression per Choi criteria in the 6 months prior to commencing axitinib therapy, were enrolled to receive 5mg axitinib twice daily until progression or toxicity (Table 3). Of the 17 patients, 4 (23.5%) had a histopathological diagnosis of high-grade/dedifferentiated SFT with the remaining 13 (76.5%) classified as metastatic SFT. Eight of the 17 (47%) patients had received previous lines of therapy, including pazopanib (7 of 17) and sunitinib (2 of 17). The primary endpoint of the study was objective response rate based on Choi criteria and the authors reported that 7 of 17 patients (41%) had a partial response as their best observed response, 6 (35%) had stable disease, and 4 had progressive disease (23%). Interestingly, 4 of the 7 (57.1%) patients pre-treated with pazopanib had a partial response to axitinib. Of note, none of the 4 patients with high grade/dedifferentiated SFT responded to axitinib.

This trial showed good antitumor activity of axitinib in metastatic SFT. Notably, over half of the patients who were pre-treated with pazopanib obtained a partial response upon subsequent treatment with axitinib. This highlights the potential for axitinib to play a role in the multi-line treatment of metastatic SFT following pazopanib failure. The apparent lack of activity in high-grade/dedifferentiated SFT suggests that the biology regulating axitinib response in SFT varies with grade. A better understanding of the biological factors driving axitinib response will not only shed light on the mechanisms of drug resistance in high-grade/dedifferentiated SFTs but also highlight candidate biomarkers of drug response.

## 9. Cediranib

Cediranib (Recentin®/AZD2171) has been evaluated in a number of preclinical models of pediatric sarcomas including MRT and RMS [64,102,103]. In these studies, cediranib displayed negligible efficacy in *in vitro* sarcoma cell line models that were tested but was observed to induce moderate reductions in *in vivo* tumor growth, with notable tumor regression observed in the rhabdoid tumor xenograft model KT-16 [102,103]. Later studies have shown cediranib to possess antiproliferative effects in cell line models of MRT, SS, and LMS [64].

Cediranib has been evaluated in several clinical trials in ASPS following the reports of activity in a small series of ASPS patients treated within a larger phase II trial conducted primarily in GIST (Table 3) [104,105]. Kummar et al. conducted an open-label, single-arm, phase II trial of cediranib in patients with metastatic ASPS not amenable to surgery, with no restrictions on prior lines of treatment (NCT00942877) [106]. Forty-six patients with histologically confirmed ASPS were enrolled onto the study, with 28 of the 46 (61%) having received prior systemic therapy, including 12 (26%) who received previous antiangiogenic therapy. Treatment efficacy was assessed by cross-sectional imaging and effect on tumor size determined by RECIST, with 43 patients evaluable for response. Of the 43 patients, 15 (35%) demonstrated a partial response to cediranib and a further 26 (60%) had a stable disease as best response. The context of these results is important, as the CBR of 95% is superior to historical reports of various cytotoxic chemotherapy schedules in metastatic ASPS demonstrating a CBR of between 31% and 80.9% [107–109]. From the trial performed by Kummar et al., pre- and post-treatment biopsies were also available for gene expression analysis by microarray, with the angiopoietin-2 (*ANGPT2)*, VEGFR1 *(FLT1)*, glutamate carboxypeptidase II (*FOLH1*), and atypical chemokine receptor 3 (*ACKR3)* genes all downregulated following treatment with cediranib. Validation by RT-PCR confirmed the downregulation of *ANGPT2, FLT1*, and *FOLH1*, as well as endothelial cell-specific molecule 1 (*ESM1*) and lysine demethylases (*KDM*), in response to cediranib. *ANGPT2, FLT1*, and *ESM1* are pro-angiogenic genes, with *ANGPT2* and *FLT1* playing a role in enhancing sprouting angiogenesis, and *ESM1*being upregulated in hypervascularised cancers [110,111]. Upregulation of *FOLH1* is associated with increased cellular proliferation in cancer models and is found in the vasculature of many tumors, whilst *KDM* are modulators of histone methylation and important epigenetic regulators [112,113]. Downregulation of these genes following cediranib provides evidence of the on-target effect of this drug through the blockade of pro-angiogenic and pro-proliferative signaling pathways which provides mechanistic insights into the molecular basis for cediranib activity.

Following on from this single-arm, phase II study, an international, multi-center, double-blinded, placebo-controlled, randomized, phase II trial of cediranib in the treatment of patients with ASPS (CASPS) was undertaken by Judson et al. (NCT01337401) [114]. Patients over the age of 16 years were enrolled and were required to have measurable metastatic disease with evidence of progression based upon RECIST in the preceding six months. Participants were randomized 2:1 to either 30mg cediranib orally daily or matched placebo. The primary end-point of this trial was the median percentage change in sum of target lesion diameters from baseline to week 24, or progression if sooner, and the results showed a significant decrease in tumor size in patients on cediranib compared to the placebo group (−8.3% vs +13.4%, p = 0.0010). Six of 31 patients (19%) in the cediranib arm had a partial response as their best response, compared to none in the placebo group (p = 0.072), with a median response duration of 16 months. PFS analysis revealed no significant difference between the two cohorts (12-month PFS 38.7% in cediranib group vs 34.4% in placebo, p = 0.28) although this was likely confounded by crossover of patients from the placebo arm to cediranib after week 24. Median OS in the cediranib arm was 27.8 months and in the placebo arm, the median has not yet been reached. Of note, when published, the median OS of the placebo arm will also likely be confounded by treatment group crossover, thereby limiting comparability between the two study arms.

Along with the study by Kummar et al., Judson et al. have confirmed the activity of cediranib in advanced, metastatic ASPS. The CASPS trial represents an important step in improving outcomes in patients with ASPS, as well as demonstrating the ability to undertake randomized, multi-center, collaborative trials in rare sarcoma subtypes. There is a need to further understand the biology of ASPS response to cediranib to shed light on the mechanisms driving both primary and acquired resistance observed in the CASPS trial. This understanding will offer further insights into strategies to overcome resistance either through the use of combination or salvage therapies with further lines of alternative TKIs. Of interest, the subset of patients who enrolled in the CASPS trial with prior exposure to TKI therapy, aside from those pre-treated with crizotinib, appeared to have equal outcomes to those without prior TKI exposure.

Looking to the future, the role of the immune system and immunomodulating therapies in the treatment of ASPS is exciting. Preclinical studies in a mouse model of ASPS have demonstrated the upregulation of monocarboxylate transporter 1 (*SLC16A1*) and basigin *(BSG*), both associated with the importation of lactate into the cells, and the downregulation of monocarboxylate transporter 4 (*SLC16A3*), a gene associated with lactate export [115]. As well as stimulating cell proliferation and angiogenesis, the excess intracellular lactate is converted to pyruvate that leads to the upregulation of hypoxia-inducible factor (HIF). Not only does HIF activate VEGF transcription, but upregulation of HIF results in the accumulation of regulatory T-cells in the tumor microenvironment, leading to T-cell suppression and heightened immune system evasion [116]. As such, the question remains whether part of the response seen with cediranib and other antiangiogenic therapies is associated with improved immune activity through the downregulation of suppressive regulatory T-cells by VEGFR targeting. The recent trial of axitinib with the anti-programmed-death-1 checkpoint inhibitor pembrolizumab lends support to the combination of antiangiogenic therapy with immune checkpoint inhibition, with promising activity demonstrated particularly in ASPS (NCT02636725) [117]. Moving forward, through a deeper understanding of the tumor immune microenvironment and its association with antiangiogenic therapy in ASPS, we may be able to develop rational combinational therapies which leverage on this interaction to provide patients with better treatments.

## 10. Nintedanib

Nintedanib (Ofev®/Vargatef®/BIBF 1120) has shown preclinical activity in a range of STS subtypes including MRT, SS, and MPNST, most of which harbor overexpression of kinases targeted by nintedanib [64,118,119]. For instance, nintedanib was found to decrease cellular proliferation of MPNST and SS cell lines, both of which express relatively high levels of PDGFR and FGFR RTKs [64,118]. This reduction in growth was associated with inhibition of PDGFR and FGFR phosphorylation and downstream AKT and/or ERK signaling, which was not observed in nintedanib-resistant Ewing's sarcoma cell lines [118]. These properties of nintedanib were also observed *in vivo* in a SS xenograft model, with an associated decrease in tumor microvessel area [118]. Combination therapy utilizing AKT and MEK inhibitors was able to phenocopy the effects of nintedanib, thereby confirming the importance of dual blockade of the AKT and ERK signaling as a means of inhibiting growth of SS and MPNST cells [118]. This study also found that nintedanib confers its antiproliferative and downstream inhibitory effects through dual inhibition of PDGFR and FGFR, as monotherapy using an FGFR inhibitor was not able to fully recapitulate the phenotype observed with nintedanib [118]. Utilizing RNA interference (RNAi), the authors showed that only the combined knockdown of FGFR1, FGFR2, and PDGFRα was able to phenocopy nintedanib treatment [118]. Similarly, nintedanib was found to display significant potency toward MRT and RMS cell lines A204 and SJCRH30, respectively, both of which overexpress PDGFR [64,119].

The EORTC Soft Tissue and Bone Sarcoma Group (STBSG) is conducting a multicenter, open-label, phase II trial randomizing advanced STS patients to receive ifosfamide or nintedanib as second-line therapy (NCT02808247, EORTC1506) [120]. Although unselective in its recruitment of STS subtypes, this trial may offer insights into the efficacy of nintedanib in STS and provide evidence for its use in the clinical setting.

## 11. Anlotinib

Anlotinib (AL3818) is a multi-target TKI that has only recently been developed and, as a result, published preclinical studies of anlotinib in STS are limited. In addition to its ability to block the activation of angiogenic and tumorigenic RTKs, it has been shown that anlotinib reduces SS cellular proliferation and xenograft tumor growth through targeting of GINS1, a DNA replication complex subunit found to be highly expressed in SS and associated with poor prognosis [121]. RNAi-mediated knockdown of *GINS1* was able to phenocopy the antiproliferative effects of anlotinib in SS cell lines, thereby confirming that the targeting of GINS1 by anlotinib was essential in achieving its antitumor effect [121]. Further preclinical studies into anlotinib may be useful in identifying additional STS subtypes that may benefit clinically from treatment with this TKI.

A phase II clinical trial of anlotinib has been completed (see Table 2) and this TKI is currently undergoing phase III evaluation in advanced STS [122,123]. Chi et al. reported data from their multi-center, single-arm, phase II study of anlotinib in antiangiogenic therapy-naïve patients with metastatic STS that had progressed on first-line anthracycline therapy (NCT02449343) [122]. They enrolled 166 patients with a broad range of STS subtypes, including LMS, LPS, SS, undifferentiated pleomorphic sarcoma, ASPS, clear cell sarcoma (CCS), and a further subgroup of other sarcomas. In this trial, anlotinib demonstrated broad-spectrum antitumor activity in chemotherapy-refractory STS, with disease control achieved in 74% of patients (107 of 166); mPFS was 5.6 months and median OS of 12 months. The context of these data are promising, particularly given the historical survival data of chemotherapy-refractory STS, such as the placebo arm of the PALETTE trial which reported an mPFS of 1.6 months and median OS of 10.7 months [13]. Such comparisons are of course limited given the heterogeneity of clinical behavior in STS; however, this does suggest that anlotinib is a promising agent in advanced STS. Interestingly, in the ASPS subgroup, a sarcoma subtype particularly resistant to cytotoxic chemotherapy, 6 of the 13 patients (46%) had a partial response to anlotinib per RECIST, with a cohort mPFS of 21 months.

The promising data from this phase II trial has led to an ongoing phase III, anlotinib in metastatic or advanced ASPS, LMS, and SS (APROMISS, NCT03016819) trial which aims to recruit 95 patients with SS and 68 with LMS who will be randomized 2:1 to anlotinib or dacarbazine, with a further 56 patients with ASPS to receive open-label anlotinib [123]. APROMISS is currently the only phase III trial currently evaluating the efficacy of a TKI across a number of different STS subtypes. Should the promising efficacy signals detected in the phase II trial translate into definitive data in the APROMISS trial, the sarcoma community may well have another TKI option for use as part of the therapeutic arsenal in advanced STS.

## 12. Sitravatinib

The published preclinical evaluation of sitravatinib (MGCD516) in STS is limited to a single publication [19]. This study reports potent inhibition of proliferation in dedifferentiated-LPS and MPNST cell lines upon sitravatinib treatment, with associated blockade of PDGFRβ, MET, and insulin-like growth factor 1 receptor (IGF1R) phosphorylation, as well as downstream AKT signaling [19]. This significant reduction in LPS growth *in vitro* is important as there are currently no TKIs approved for use in this STS subtype. In the LPS and MPNST cell lines assessed, sitravatinib displayed greater antiproliferative effects compared to pazopanib, crizotinib, and imatinib, with an associated increased reduction in RTK and AKT phosphorylation both *in vitro* and *in vivo* [19]. To determine if the antiproliferative effects observed in cells were due to the inhibition of RTKs by sitravatinib, the authors utilized siRNA-mediated knockdown of PDGFRβ, MET, IGF1R, and KIT to phenocopy sitravatinib’s effects [19]. The antiproliferative effect induced by silencing multiple RTKs simultaneously was comparable to those observed with sitravatinib, thereby confirming the correlation between inhibition of these RTKs and the significant reduction in tumor cell proliferation [19].

The efficacy of sitravatinib in LPS in the preclinical setting has been translated into an ongoing phase II clinical trial in well-differentiated/dedifferentiated-LPS, as well as other advanced sarcomas (NCT02978859) [124,125]. This prospective, open-label, single-arm, phase II study is currently enrolling a target of 29 patients under a Simon II stage design and the study is expected to complete in January 2021 [124,125]. The first stage of the study will recruit 13 patients with a diagnosis of progressive well-differentiated or dedifferentiated-LPS to receive 150mg of oral sitravatinib daily, with PFS at 12 weeks as the primary endpoint. Interim analysis will determine efficacy and, if satisfactory, the second stage of the trial will involve enrollment of a further 16 patients with well-differentiated or dedifferentiated-LPS. If the Simon II stage design fails, the next 16 patients enrolled will be made up of cohorts of 4 patients each, with a diagnosis of MPNST, SS, aRMS, and ASPS. Due to the lack of demonstrated efficacy in LPS in a number of previous clinical trials involving TKIs, this trial represents an important opportunity toward identifying an effective treatment for these patients.

## 13. Crizotinib

Crizotinib (Xalkori®/PF-02341066) is a multi-target TKI that inhibits the anaplastic lymphoma kinase (ALK) and MET signaling pathways. It has shown antitumor effects in models of small round cell tumors, SS, and aRMS. Utilizing a 119 anticancer inhibitor screen, crizotinib was found to be the only TKI that resulted in significant suppression of cellular growth in patient-derived *CIC-DUX4* fusion-positive small round cell tumor primary cells [126]. In another study, a panel of SS cell lines were subjected to phosphoproteomic profiling and ALK was shown to be an oncogenic driver in a subset of cell lines [127,128]. SS cell lines were therefore subjected to escalating doses of crizotinib treatment and only those lines found to highly express either ALK or MET displayed significant sensitivity to the drug [64,127]. The observed decrease in cell proliferation was coupled with a reduction in downstream ERK, AKT, and STAT3 phosphorylation, as well as induction of G1 cell cycle arrest and apoptosis [127]. Xenograft models of ALK- and MET-dependent SS cells also displayed sensitivity to crizotinib which resulted in durable tumor regression alongside a significant reduction in microvessel area [127]. In another study, it was demonstrated that ALK and MET-expressing aRMS cell lines were sensitive to crizotinib and that this drug inhibited cell migration and invasiveness [129].

The EORTC STBSG-sponsored CREATE trial was an international, biomarker-driven, single-arm, non-randomized, open-label, phase II trial with the aim of assessing the efficacy and safety of crizotinib in ASPS, inflammatory myofibroblastic tumors (IMT), CCS, and aRMS (NCT01524926, EORTC90101) (Table 3) [130–132]. These sarcoma subtypes were chosen as they are known to harbor specific alterations that result in ALK and/or MET activation. All the patients enrolled received 250mg crizotinib orally twice daily without masking or randomization. The primary end-point across all cohorts was objective response rate as determined by RECIST on cross-sectional imaging (Table 2).

The rationale for including a cohort of ASPS in the trial was driven by the characteristic chromosomal translocation seen in this subtype which comprises of a fusion of the transcription factor E3 (*TFE3*) gene to the *ASPCR1* gene. The resulting chimeric transcription factor leads to overexpression of MET [133]. The ASPS cohort in CREATE consisted of 48 patients with metastatic or advanced ASPS not amenable to routine curative management, of which 45 were available for assessment of crizotinib activity [130]. Twenty-five of the 48 (52.1%) patients had no previous systemic anticancer therapy. The best observed responses were 2 (4.4%) partial responses, 39 (86.7%) with stable disease, and 4 (8.9%) with progressive disease. Six of the 48 patients (12.5%) suffered grade 3/4 toxicities.

Approximately 50% of IMTs are known to harbor *ALK* gene rearrangements, predominantly translocations with variable fusion partners, resulting in the overexpression of chimeric ALK protein. The IMT cohort in CREATE consisted of 20 patients with advanced IMT deemed incurable through routine management options and 19 of those enrolled were available for assessment of efficacy [131]. The presence of *ALK* gene rearrangement was determined centrally using immunohistochemistry and FISH techniques and deemed positive if greater than 15% of cells demonstrated confirmed gene rearrangements on FISH analysis or positive staining for ALK on immunohistochemistry. In the cases which harbored the *ALK* fusion, 6 of 12 (50%) patients achieved an objective response to crizotinib, compared to only 1 of 7 (14.3%) patients with unaltered ALK. In terms of toxicity, 8 serious adverse events related to crizotinib were observed in 5 patients (25%). With an objective response observed in half of IMT patients with a proven rearrangement of *ALK*, the CREATE trial supports the use of crizotinib in this clinical setting [131].

CCS is a sarcoma affecting tendons and aponeuroses and is characterized by a chromosomal translocation resulting in the generation of a *EWSR1-ATF1* fusion gene and subsequent aberrant overexpression of MET [134]. For the CCS cohort in CREATE, 34 patients with a centrally confirmed diagnosis of CCS were enrolled onto the study, of which 28 were assessable for response [132]. Presence of the *EWSR1-ATF1* fusion gene was confirmed through FISH analysis, with a minimum of 15% of cells required to demonstrate the *EWSR1-ATF1* fusion gene for the case to be deemed positive for MET amplification. Twenty-five of the 34 (73.5%) patients had not received prior systemic therapy. Partial response was observed in 1 of 26 (3.8%) patients, with stable disease observed in 17 (65.4%) and progressive disease in the remaining 8 (30.8%) patients. The mPFS observed in this cohort of 4.4 months is favorable compared to previously published data reporting a mPFS of 2.6 months in patients with CCS treated with first-line cytotoxic chemotherapy [135].

The CREATE trial is an example of a biomarker-driven basket trial, leveraging on the demonstrated biological activity of crizotinib in preclinical work and applying that to sarcoma subtypes with known genetic alterations resulting in the upregulation of ALK and/or MET. This trial has simultaneously identified a novel targeted therapy with clinical efficacy in multiple STS subtypes and is a good model for biomarker or genotype-driven trial designs for the future evaluation of TKIs in non-GIST STS.

## 14. Dasatinib

Promising preclinical results in a variety of STS subtypes has revealed a potential emerging role of dasatinib (Sprycel®/BMS-354,825) in the evolving landscape of contemporary STS treatment. For instance, dasatinib significantly inhibited the growth of CRKL-dependent embryonal RMS and aRMS cell line and xenograft models through inhibition of the Src-family kinases, which are associated regulators of CRKL activity [136]. Dasatinib has also been shown to block tumor cell growth by directly repressing Ephrin B4 receptor and PDGFRβ phosphorylation in primary cell and allograft models of aRMS [137]. Similar antiproliferative effects have been observed in SS, ASPS, LPS, aRMS, and MRT preclinical models, with direct inhibition of Src and/or PDGFRα [64,65,138–140]. Within these models, dasatinib was also found to induce apoptosis and cell cycle arrest, with concomitant inhibition of cellular migration and invasiveness [137–141]. Additionally, dasatinib sensitivity has also been reported in cell line models of fibrosarcoma, MPNST, RMS, spindle cell sarcoma, epithelioid sarcoma, and LMS [64]. Furthermore, a recent preclinical study has reported the activity of dasatinib in a panel of patient-derived sarcoma cells that harbor a broad range of translocations [141].

Despite the promising potency of dasatinib in a broad range of preclinical models, the efficacy of this drug in the clinical setting has largely been disappointing. Dasatinib has been evaluated in an open-label, single-arm, phase II trial in ASPS, chondrosarcoma, chordoma, epithelioid sarcoma, and SFT (NCT00464620, SARC009) (Table 2) [142]. These subtypes were selected due to their indolent nature and the lack of effective therapies in cases with unresectable or metastatic lesions. Eligibility criteria included patients over the age of 13 years, a diagnosis of ASPS or grade 1/2 for the other subtypes, a measurable lesion on cross-sectional imaging, and tumors incurable using conventional therapies. Each patient was treated with dasatinib at a dose of 100mg twice daily. One hundred and nine patients were recruited to the study, composed of 12 patients with ASPS (11%), 33 (30%) with chondrosarcoma, 32 (29%) with chordoma, 7 (6%) with epithelioid sarcoma, and 25 (23%) with SFT. The overall rate of 6 month PFS by Choi criteria was 48%, falling short of the trial’s stated primary end-point of achieving disease control at 6 months in at least 50% of the recruited patients. There was considerable between-subtype variation, with the rate of PFS at 6 months of 62% in the ASPS cohort, 57% in epithelioid sarcoma, 54% in chordoma, 47% in chondrosarcoma, and lowest in the SFT cohort at 30% (Table 3). Of note, 18% of patients with chondrosarcoma or chordoma, both known to be chemoresistant, were seen to have an objective response to dasatinib on cross-sectional imaging as per Choi criteria. Across the whole cohort, a median of 4 cycles of dasatinib were administered with treatment interruption necessary due to toxicity in 62 of the 109 patients (57%) and a dose reduction in 36 (33%) patients.

Based on this study, dasatinib failed to demonstrate clinically meaningful antitumor effect in a number of the subgroups enrolled, most notably SFT. The lack of placebo control limits our ability to draw substantial conclusions from the results, however, based on the encouraging antitumor activity observed in ASPS, epithelioid sarcoma, and chordoma there may be a basis for further investigation of this drug in these subtypes.

## 15. NTRK inhibitors

The NTRK family consists of the neurotrophic factor receptors TRKA, TRKB, and TRKC, which play pivotal roles in physiological neuronal development and differentiation, but have also been established as oncogenic drivers in a range of human malignancies [50]. The most common mechanism of NTRK oncogenesis occurs through intra- and inter-chromosomal rearrangements resulting in constitutively active NTRK fusion proteins, some of which have been identified in STS [50]. For instance, the gene fusion, *ETV6-NTRK3*, is considered pathognomonic in infantile fibrosarcomas, with >90% incidence within this subtype [50,52].

The NTRK inhibitor larotrectinib (Vitrakvi®/LOXO-101/ARRY-470) has recently been approved by the FDA for advanced or metastatic solid tumors harboring *NTRK* gene fusions [143]. The approval was based on the findings of a clinical development program which included patients of any age and any tumor type and encompassed three clinical study protocols (NCT02122913, NCT02637687, and NCT02576431) [144]. The three clinical studies were; a safety and dose-escalation phase I study involving adults, a phase I-II study involving children with advanced solid or primary central nervous system tumors, and a single-arm, non-randomized, phase II study of adolescents and adults with *NTRK*-fusion positive tumors. A maximally tolerated dose of larotrectinib was not defined during the phase I study and the recommended dose of 100mg twice daily of larotrectinib was utilized for the phase II study. The primary end-point of the study was overall response rate, assessed by independent radiology review, and determined by RECIST. The combined program cohort of 55 patients was made up of 17 unique cancer diagnoses, including 7 cases of infantile fibrosarcoma and 11 STS of unspecified histological subtypes. The reported overall response rate was 80% (44 out of 55 patients) and was independent of tumor type, age, or type of *NTRK* fusion. mPFS had not been reached at a median follow-up of 9.9 months, nor had a median duration of response been met at a median follow-up of 8.3 months. Larotrectinib was well tolerated with a dose reduction only required in 8 of the 55 patients (15%) and no treatment-related grade 4 or 5 adverse events noted.

The significant antitumor effect observed in these trials demonstrates the rationale for undertaking biomarker focused trials against known molecular targets. The impressive overall response rate supports the use of larotrectinib in patients with sarcomas harboring *NTRK* alterations. In addition, across the three clinical trials described above, the authors were able to obtain post-treatment tumor tissue in 10 patients with disease progression following a minimum 6 months of stable disease or an objective response, with the goal of determining the mechanisms driving acquired resistance. A variety of kinase domain mutations in the *NRTK* gene were identified from these specimens. Moving forward, LOXO-195, a next-generation NTRK inhibitor specifically designed to inhibit these kinase domain mutations associated with acquired drug resistance may emerge as an important option for patients who progress on larotrectinib. LOXO-195 is currently undergoing phase I-II trials in adults and children with progressive disease following NTRK-targeted therapy (NCT03215511) [145].

## 16. Biomarkers for TKI response in STS

At present, there is an unmet clinical need for validated biomarkers predictive of response to TKIs in STS. In the grouped post-hoc analysis of the cohorts of patients treated with pazopanib in the PALETTE trial and the preceding phase II clinical trial, only performance status and tumor grade were identified as predictive of response to pazopanib [13,146]. However, these are well established prognostic factors in STS and no new biomarkers for response were identified [147]. Other clinical trials of TKIs in STS have included sample collection with the goal of biomarker identification. In patients with GIST treated with sunitinib, Deprimo et al. identified that a decreased level from baseline of soluble KIT in plasma was associated with increased time to progression, whilst Norden-Zfoni et al. showed that increased circulating endothelial cells upon treatment initiation was associated with improved outcomes [148,149]. Raut et al. identified that following initiation of sorafenib in advanced unselected soft tissue sarcomas, decreased levels of VEGFR2 following 28 days of therapy correlated with disease progression [150].

Advances in imaging technology have also paved the way for imaging modalities that are potentially able to define TKI responses more accurately and at earlier stages of treatment. The most widely utilized modality at present is positron emission tomography (PET) which enables visualization of cell processes *in vivo* through radioactive probes, most commonly ^18^F-fluorodeoxyglucose (FDG). FDG-PET has shown value as a candidate imaging biomarker in the treatment of GIST with the TKI imatinib, with Goerres et al. reporting that after one cycle of treatment, tumors without pathological FDG accumulation went on to have a longer mean overall survival when compared to tumors displaying ongoing FDG-avid areas [151]. Furthermore, Vlenterie et al. performed FDG-PET scans at baseline and 2 weeks after treatment initiation in 20 patients with unselected STS treated with pazopanib. They reported that visual response analysis of FDG-PET scans after 2 weeks of pazopanib therapy was able to classify 42% of patients as non-responders who subsequently went on to cease pazopanib at 8 weeks due to disease progression as determined by computerized tomography scan [152]. This ability to identify non-responders earlier in their treatment results in less exposure of patients to costly and potentially toxic therapies.

The progress being made in biomarker discovery is encouraging, however, the markers discussed above are yet to be validated and can only be assessed after initiation of therapy. Looking to the future, more research is necessary to discover better predictive and validated biomarkers to allow the prospective selection of patients most likely to respond to specific TKIs.

## 17. Inter-patient pharmacological variability associated with TKI therapy

Many pharmacological studies in a variety of cancer types have shown that patients treated with TKIs display high inter-patient pharmacokinetic variability [153–166]. This metabolic variation will therefore result in certain patients being under- or overdosed, when using a standard dosing regimen, leading to a lack of clinical efficacy or increased toxicity and adverse effects, respectively. In addition, many of the independent, individual covariables within a studied population (such as age, gender, body weight, and race), often show a significant inter-covariable difference in pharmacokinetics. However, these are currently not applicable to the clinical adjustment of dose for the entire population [155,158,162,163,167]. Pharmacokinetic variability has also been shown to occur over time with decreased TKI exposure being observed upon long-term treatment [157,168,169].

The observed inter-patient variability is due to numerous pharmacokinetic parameters such as cytochrome P450 (CYP) activity, drug–drug interactions (DDI), drug-transporter activity such as p-glycoprotein (PGP), and plasma protein binding. The main phase I metabolic pathway of most TKIs is through the CYP pathway, with the CYP3A4 isoenzyme accounting for the main route of metabolism for all of the TKIs discussed except for nintedanib [170–172]. Although marginally metabolized by CYP3A4, nintendanib is primarily metabolized by esterases and UDP-glucuronosyltransferases [170]. Therefore, patients with increased or decreased levels of CYP activity or those harboring CYP polymorphisms will result in substantial differences in TKI plasma concentrations [173]. In addition, TKI plasma levels can also be greatly altered due to DDI mediated by CYP activity where concomitant treatment of TKIs with CYP inhibitors or inducers can result in the decrease or increase of TKI metabolism [170,173]. Similar considerations in terms of activation levels and genetic polymorphisms must be taken into account for cellular drug efflux pumps such as PGP, for which the majority of TKIs discussed are substrates [160,164,170]. Furthermore, many of the TKIs discussed are incidental CYP and/or PGP inhibitors themselves, further exacerbating potential DDIs [170]. Finally, all of these TKIs display very high plasma protein binding (90->99%), except for larotrectinib (70%) [170,174,175]. Therefore, the vast majority of administered TKI become bound to proteins within the plasma and are unable to fulfill their biological intracellular activity. Consequently, a greater dosage of TKI needs to be administered to ensure adequate drug levels to have the desired therapeutic effect.

Therefore, there is a need for a broader discussion about the implementation of therapeutic drug monitoring (TDM) techniques for personalized TKI therapy. Several studies have highlighted how maintaining the patient plasma concentration of a TKI above a trough plasma concentration (C_min_) threshold through inter-patient and time-point specific dosage variation, whilst also maintaining a concentration below one that would result in toxicity/adverse effects, has resulted in increased molecular responses and PFS in GIST, RCC, and chronic myeloid leukemia patients [153,154,156,168,169,176,177]. For instance, in RCC, a C_min_ threshold of ≥20mg/L pazopanib has been clinically validated to provide a significant increase in PFS and tumor shrinkage when compared to patients displaying < 20mg/L C_min_ [177]. In STS, Verheijen et al. have described similar trends with an increased PFS and tumor shrinkage in patients with an average C_min_ of pazopanib at ≥20mg/L [178,179]. The 2016 Verheijen study also showed that this relationship between C_min_ and treatment response occurred in the overall population of analyzed patients with solid tumors, rather than just in the STS subset [178]. These studies both concluded that individualized pazopanib dose escalation through TDM could optimize treatment in underexposed STS patients who displayed a C_min_ of <20mg/L, thereby elevating individual C_min_ levels above this threshold to result in a greater therapeutic response [178,179].

TDM could therefore limit the possibility of sub- and supra-exposure in TKI therapy through variation of dosages on a patient-and time-specific basis to increase therapeutic activity, reducing toxicity/adverse effects, and to limit the intrinsic inter-patient response variability universally observed in TKI therapy [153,154,169,178,179]. Inter-patient pharmacokinetic variability is therefore an important consideration for clinicians in the treatment of STS patients with TKIs.

## 18. Expert opinion

The introduction of TKIs into the clinic has revolutionized the way many cancers are treated. One of the biggest challenges related to the current management of non-GIST sarcomas with TKIs is the lack of any validated predictive biomarkers. As a field, more translational research needs to be undertaken over the next five years to discover robust biomarkers to identify patients who are most likely to achieve durable benefit from TKIs. Should such biomarkers be identified, the emphasis in clinical trial design in sarcomas should move away from the ‘one size fits all’ paradigm in which heterogeneous cohorts of multiple histological subtypes in small numbers are treated with the same drug or schedule [180]. In contrast, where possible, biomarker-guided basket trials such as the CREATE trial, which evaluate multiple disease types with a common oncogenic driver matched to a specific targeted therapy, should be considered. We anticipate that moving toward biomarker-guided clinical studies in sarcoma will transform the current ‘one size fits all’ approach into a personalized medicine paradigm where the right patient is treated with the right drug at the right time. Not only will this benefit patients, through rational administration of the most effective anticancer therapies, it will also improve cost-effectiveness and quality of life measures in the management of sarcomas. Due to the rarity of sarcomas, the step from phase II to phase III trials is expensive, time consuming, and resource intensive, often requiring international collaboration over a long period to recruit sufficient numbers for an adequately powered trial. We anticipate that biomarker-guided trials will also help address the problem faced in sarcoma where a large number of phase II trials of TKIs have been conducted but relatively few placebo-controlled phase III trials.

The underlying biology driving TKI response and resistance in STS is also poorly understood and this remains an important knowledge gap to address in this field. Through the use of patient-derived preclinical models and molecular profiling of tissue specimens, it is anticipated that we will gain a better understanding of the biological factors that govern TKI response. At present, there is a paucity of clinical evidence related to the role of TKIs in the multi-line setting in non-GIST STS. In order to optimize patient management and drug selection, the role of regorafenib and other TKIs described in this review in the multi-line setting should be explored. As we develop a better understanding of the biology and mechanisms of TKI activity and acquired resistance in non-GIST STS, this knowledge will shed light on the role of sequential drug treatment and direct the development of clinical trials to evaluate multi-line TKI strategies as a means of achieving durable tumor responses in patients. The clinical experience in RCC may act as a template in this regard where the use of multiple lines of multi-target TKIs is the standard of care [181]. Indeed, evidence from the CASPS trial where patients with prior exposure to other TKIs had the same cediranib outcomes to those without prior TKI exposure suggests that selected STS subtypes may similarly benefit from such a multi-line strategy [114]. Another important area to consider is that a standard dosing regimen of TKI may not be a therapeutically beneficial strategy for many patients due to the high inter-patient variability that exists in TKI pharmacokinetics and pharmacodynamics. There is therefore an increasing movement towards utilizing TDM where patient-specific TKI doses are administered over time, resulting in optimal TKI exposure. TKIs represent an exciting shift in the paradigm in the treatment of multiple cancer types. However, there is much progress to be made in STS before similar benefits can be achieved in this group of rare cancers. Over the next five years, we envisage that the number of TKIs licensed for use in STS is likely to increase, which will offer new hope for patients with these cancers with poor outcomes in the advanced disease setting. However, we also anticipate that such TKIs will face similar issues as those encountered with pazopanib, namely drug resistance and heterogeneity in patient response. As our understanding of the biology driving TKI therapeutic response improves, the sarcoma community will need to identify predictive biomarkers that will enable TKI regimens to be matched to individual patients. This deeper biological understanding will also define the role of sequential TKI therapies in the management of STS, providing clinicians with salvage options following failure of first-line TKI therapy. The next five years offers the sarcoma research community an exciting opportunity to take great strides forward in defining the role TKIs in the management of STS and improving long-term survival in patients.

## 19. Conclusion

The role of TKIs in the treatment of sarcomas continues to expand with recent positive trials such as crizotinib in IMT (CREATE), cediranib in ASPS (CASPS), and sorefanib in desmoid tumors (ALLIANCE A091105). Ongoing phase III trials such as APROMISS highlight the potential that additional TKI options are on the horizon for non-GIST STS. As our knowledge of the biology underlying response and resistance in TKIs increases, our ability to develop patient-specific therapies and multi-line treatment strategies will improve. To drive this promising area of research forward, the research and medical communities must continue to come together to collaborate on large-scale trials of the most promising agents in this rare group of cancers to ensure they make the transition from bench to bedside.

## Article highlights

- Soft tissue sarcomas (STS) are a highly heterogeneous group of cancers comprising over 50 different histological subtypes that display contrasting responses to systemic therapy.
- Tyrosine kinase inhibitors (TKIs) have the potential to become an increasingly important component in the arsenal of targeted therapies to treat STS for which they have shown promising preclinical activity.
- Pazopanib is currently the only TKI approved for use in advanced STS, with associated issues concerning clinical efficacy, overall survival benefit, and drug resistance, thereby highlighting the unmet need for novel therapeutic strategies in improving STS therapy.
- The phase III trial of sorafenib in desmoid tumors provides evidence of TKI activity in this soft tissue tumor, with durable benefit seen in a substantial proportion of patients.
- The CASPS international, phase III trial of cediranib in alveolar soft part sarcoma presents high-quality evidence of efficacy of a TKI in this rare disease, with a significant decrease in tumor size in the cediranib treatment arm versus placebo.
- In solitary fibrous tumors, axitinib represents a promising potential compound for further exploration, demonstrating activity in progressive or malignant cases including those patients pre-treated with pazopanib, but showing inactivity in high grade/differentiated cases.
- The basket-type trials of larotrectinib show the promise of biomarker-driven trials and represent an exciting opportunity to embed patients with sarcomas within trials of focused targeted therapies.
- Phase III clinical trials in sarcoma are rare and a large proportion of clinical advances related to TKI therapy is based upon smaller phase II trials or retrospective series. Moving forward, the focus should be on identifying promising compounds and supporting multi-center collaborative phase III trials.
